# Polymorphisms of Pre-miR-499 rs3746444 T/C and Pre-miR-146a rs2910164 C/G in the Autoimmune Diseases of Rheumatoid Arthritis and Systemic Lupus Erythematosus in the West of Iran

**Published:** 2020-04

**Authors:** Kolsoum AHMADI, Azam SOLEIMANI, Shadi SOLEIMANI MOTLAGH, Shahram BAHARVAND AHMADI, Mohammad ALMASIAN, Ali Asghar KIANI

**Affiliations:** 1. Razi Herbal Medicines Research Center, Lorestan University of Medical Sciences, Khorramabad, Iran; 2. Department of Biology, Science and Research Branch, Islamic Azad University, Tehran, Iran; 3. Student Research Committee, Lorestan University of Medical Sciences, Khorramabad, Iran; 4. Department of Internal Medicine, Artesh University of Medical Sciences, Tehran, Iran; 5. School of Medicine, Lorestan University of Medical Sciences, Khorramabad, Iran; 6. Department of Hematology and Blood Transfusion, School of Allied Medical Sciences, Lorestan University of Medical Sciences, Khorramabad, Iran

**Keywords:** MicroRNA polymorphisms, Rheumatoid arthritis, Systemic lupus erythematosus

## Abstract

**Background:**

The present research is a case-control study to analyze the influence of pre-miRNA-146a rs2910164 and pre-miRNA-499 rs3746444 polymorphisms as candidate susceptibility factors for both rheumatoid arthritis (RA) and systemic lupus erythematosus (SLE).

**Methods:**

Polymorphism in miR146 and miR499 using ARMS-PCR was genotyped on 139 autoimmune disease (AD) patients (89 RA and 50 SLE) referred to Educational Hospitals of Khorramabad, Lorestan Province, west of Iran in 2018–2019 and 237 healthy control subjects.

**Results:**

A significant increase in the likelihood of carrying the GC vs. GG of pre-miR146-rs2910164 and T vs C allele of pre-miR499- rs3746444 in patients with RA was found. On the contrary, patients with RA were less likely to carry the TC + CC vs TT genotype and the C vs T allele of pre-miR499- rs374644. In females with the GC vs GG and GC+ CC vs GG genotypes, a significant association was found with the increased risk of RA. Interestingly, the genotypic combination of TC of the pre-miR499-rs374644 with GG of pre-miR146-rs2910164 more strongly decreased the risk of RA. In patients with SLE, no notable associations were found between both pre-miRNA-146a rs2910164 and pre-miRNA-499 rs3746444 with risk of disease.

**Conclusion:**

Genetic polymorphisms of miR146 rs2910164 is associated with RA susceptibility especially in females. Interestingly, there is a potential in miR499 to reduce the risk with the protective effect of gene-gene interactions on miR146 in RA disease.

## Introduction

Autoimmune diseases (ADs) are complicated conditions triggered by loss of tolerance to self-antigens, leading to immune-mediated tissue destruction and/or multiple organ failures (1). World-wide, ADs are prevalent among 8% of the population, 78% of whom are women (2,3). Increasing evidence obtained from laboratory investigations has shown that miRNAs and single nucleotide polymorphisms within them are involved in the regulation of the expression of numerous genes, playing a key role in normal immune responses and the pathogenesis of inflammatory and autoimmune diseases (4–6). Some miR-polymorphisms and mutations are able to directly interfere with both the binding to target and the function of miRNAs (7,8). Molecular epidemiological studies have examined the association among the variants of pre-miRNA-146a rs2910164 and pre-miRNA-499 rs3746444 with susceptibility to many diseases such as ADs, including RA and SLE; however, the results are still highly debatable (9–17).

Rs2910164 G>C polymorphism is located in the precursor stem region of the miR-146a gene in the human chromosome 5q33.3 and leads to a change from the G:U pair to the C:U mismatch in its stem structure (18)Rs3746444 T/C polymorphism is located in the stem region of miR-499 in the human chromosome at position 20q11.22 and leads to a change from the A:U pair to the G:U mismatch in its stem structure (19).

This study examined the association between the two common single nucleotide polymorphisms (SNPs) of pre-miR-499 rs3746444 T/C and pre-miR-146a rs2910164 C/G with the risk of developing RA and SLE in an Iranian population.

## Materials and Methods

### Participants

The case group was selected in between patients with systemic lupus erythematosus and rheumatoid arthritis, based on rheumatologist’s diagnosis and clinical trials. Sample of patients who did not have sufficient DNA for PCR reaction or complete clinical records were excluded from the study. Patients were also entered to study according to the new classification criteria of ACR-EULAR 2010. The group of control were individuals who did not have any autoimmune disease in themselves or even their first-degree relatives. Moreover, participation for all subjects in this study were voluntarily and with informed consent. This case-control study consisted of 139 autoimmune patients (89 RA and 50 SLE) referred to Educational Hospitals of Khorramabad, Lorestan Province, west of Iran in 2018–2019 and 237 healthy control participants. All control participants were unrelated to patients, but were from the same geographical origin and lived in the same region as the patients with RA and SLE.

The Ethics Committee of the Lorestan University of Medical Sciences approved the project with the approval number of LUMS.REC.1396.242 and informed consents were obtained from all participants.

### Genotyping assay

DNA extraction from whole blood samples was carried out using the Boiling method. The genotyping of pre-miRNA-146a rs2910164 and pre-miRNA-499 rs3746444 was done using the Tetra amplification refractory mutation system-polymerase chain reaction (T-ARMS-PCR). In the present study, primers and T-ARMS-PCR conditions used were set based on the protocols (9) (Table 1). PCR reaction was done with initial denaturation at 95 °C for 5 min in 30 cycles of 30 sec at 95 °C, annealing at 25 sec in 61 °C for rs2910164 SNP, 27 sec at 60 °C for rs3746444 SNP and 25 sec at 72 °C, and final extension at 72 °C for 10 min. Finally, PCR products for detection genotypes were loaded using electrophoresis with 2% agarose in the gel. As Shown in Fig. 1 (a, b), the sizes of the amplified fragments of the rs2910164 were 364 bp for the control band, 169 bp for the C allele and 249 bp for the G allele. The fragment sizes for rs3746444 were 206 bp for the C allele, 268 bp for the T allele, and 422 bp for the two outer primers (the control band). To ensure the quality of the genotyping, 20% of the samples were randomly re-digested and the preliminary results were confirmed.

**Fig. 1: a.**
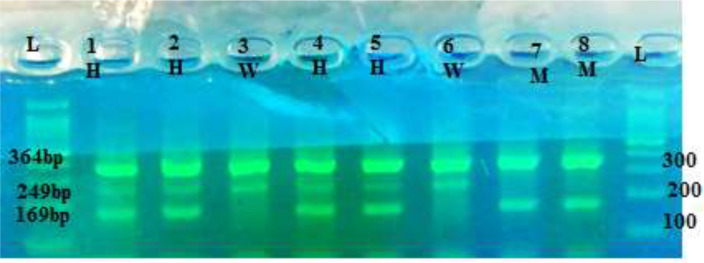
The SNP of pre-miRNA-146a(rs2910164). L:Ladder; lanes 3 and 6 wild type (W) homozygouse allels (GG); lanes 1, 2, 4 and 5 heterozygouse(H) allels (GC); lane 7and 8 mutant (M)homozygouse allels (CC)

**Fig. 1: b.**
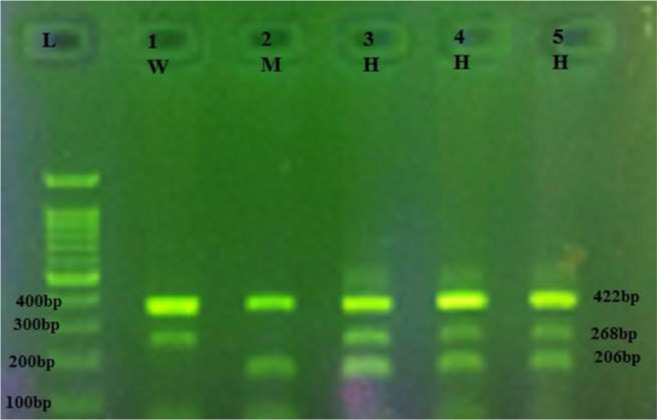
The SNP of pre-miRNA-499 (rs3746444). L:Ladder; lane 1 wild type (W) homozygouse allels (TT); lanes 3, 4 and 5 heterozygouse(H) allels (TC); lane 2 mutant (M)homozygouse allels (CC)

**Table 1:** Primers sequence of the current study

***Gene***	***Primer***	***Sequence (5′-3′)***	***Amplicon size (bp)***
miR-146a; rs2910164 G>C	FO	GGCCTGGTCTCCTCCAGATGTTTAT	364
	RO	ATACCTTCAGAGCCTGAGACTCTGCC	364
	FI (C allele)	ATGGGTTGTGTCAGTGTCAGACGTC	169
	RI (G allele)	GATATCCCAGCTGAAGAACTGAATTTGAC	249
miR-499; rs3746444 T>C	FO	GAGTGACCAGGCCCCTTGTCTCTATTAG	422
	RO	TTGCTCTTTCACTCTCATTCTGGTGATG	422
	FI (C allele)	ATGTTTAACTCCTCTCCACGTGACCG	206
	RI (T allele	GGGAAGCAGCACAGACTTGCTGTTAT	268

FO, forward outer; RO, reverse outer; FI, forward inner; RI, reverse inner (9)

### Statistical Analysis

For statistical analysis, data were entered into the SPSS application Ver. 18.0 (Chicago, IL, USA). The significance level of the genotypes were checked by the Pearson chi-square test. Crosstabs were used to calculate the risks of developing RA and SLE in the participants by estimating the odds ratio (OR) and the 95% confidence interval (CI). To analyze the performed adjustments made for the frequency of genotypes, gene–gene interaction and sex in patients were compared with the control group. *P*-values less than 0.05 were considered statistically significant.

## Results

### Characteristics of the study population

Out of the 89 RA patients who participated in this study 65 (73.03%) were female and 24 (26.96%) were male. Additionally, from among 50 SLE patients, 46 (92%) were female and 4 (8%) were male. 138 (58.22%) females and 99 (41.77%) males were enrolled in the control (healthy) group of the study. The average ages of the participants of the RA and SLE groups were 48.2 and 45.22 yr respectively and it was 43.84 in the control group. The frequency of genotypes was studied with the Hardy-Weinberg equilibrium for the control group.

### The Association between pre-miRNA-146a rs2910164 Polymorphism and the Risk of Developing RA and SLE

In the analysis of *rs2910164* genotypes, the GC of (Codominani Model) and GG +CC vs GG genotypes (dominani Model) were related to increased risk of developing RA (GC vs GG: *P*= 0.02, OR=1.83, 95% CI=1.09–3.08; GG +CC vs GG: *P*= 0.05, OR=1.62, 95% CI=0.98–2.68, respectively). However, there was no effect on the risk of developing SLE and on the probability of the simultaneous occurrence of both RA and SLE (Table 2).

**Table 2:** miR146rs2910164 genotypes and allele frequency in the rheumatoid arthritis and systemic lupus erythematosus patients and control participants

***Model***	***miR146 rs2910164***	***Control (237) n (%)***	***RA (89) n (%)***	***P-value OR (CI 95%)***	***SLE (50) n (%)***	***P- value OR (CI 95%)***	***RA + SLE (139) n (%)***	**P- *value OR (CI 95%)***
Codominant	GG	113(47.6)	32(35.96)	Ref	28(56)	Ref	60(43.2)	Ref
	GC	98(41.4)	51(57.3)	0.02	18(36)	0.36	69(49.6)	0.20
				1.83 (1.09–3.08)		0.74 (0.38–1.42)		1.32 (0.85–2.05)
	CC	26(11)	6(6.74)	0.68	4(8)	0.40	10(7.2)	0.42
				0.81 (0.30–2.15)		0.62 (0.20–1.92)		0.72 (0.32–1.60)
Dominant	GG	113 (47.6)	32(35.96)	Ref	28(56)	Ref	60(43.2)	Ref
	GC+ CC	124(52.4)	57(64.04)	0.057	22(44)	0.28	79(56.8)	0.39
				1.62(0.98–2.68)		0.71(0.38–1.32)		1.19(0.78–1.82)
Recessive	GG+ GC	211(89)	83(93.26)	Ref	46(92)	Ref	129(92.8)	Ref
	CC	26 (11)	6(6.74)	0.25	4(8)	0.53	10(7.2)	0.23
				0.58(0.23–1.47)		0.70(0.23–2.11)		0.62(0.29–1.34)
Alleles	G	324(68.35)	115(64.6)	Ref	74(74)	Ref	189(68)	Ref
	C	150(31.65)	63(35.4)	0.36	26(26)	0.26	89(32)	0.92
				1.18(0.82–1.70)		0.75(0.46–1.23)		1.01(0.74–1.39)

### Association between pre-miRNA-499 rs3746444 Polymorphism and Risk of Developing RA and SLE

In the recessive model, a significant increase in the risk of carrying the T vs C allele of pre-miR499 rs3746444 in patients with RA was found. However, a significant risk reduction was found between TC+CC vs TT genotype of pre-miR499 rs3746444 (Dominani Model) and C vs T allele in RA patients (TC + CC vs TT: *P*=0.028, OR=0.54, 95% CI=0.31–0.94; C vs T, *P*=0.01, OR=0.54, 95% CI=0.33–0.89, respectively). Moreover, in this analysis, no increased risks were observed for developing SLE or for developing both RA and SLE simultaneously (Table 3).

**Table 3:** miR499rs3746444 genotypes and allele frequency in the rheumatoid arthritis and systemic lupus erythematosus patients and control participants

***Model***	***miR499 rs3746444***	***Control(237) n (%)***	***RA (89) n (%)***	**P-*value OR (CI 95%)***	***SLE (50) n (%)***	**P-*value OR (CI 95%)***	***RA + SLE (139) n (%)***	**P-*value OR (CI 95%)***
Codominant	TT	145(61.2)	66(74.2)	Ref	27(54)	Ref	93(66.9)	Ref
	TC	83(35)	23(25.8)	0.07	23(46)	0.20	46(33.1)	0.51
				0.60(0.35–1.05)		1.48(0.80–2.76)		0.86(0.55–1.34)
	CC	9(3.8)	0	0.06	0	0.35	0	0.028
				0		0		0
Dominant	TT	145(61.2)	66(74.2)	Ref	27(54)	Ref	93(66.9)	Ref
	TC + CC	92(38.8)	23(25.8)	0.028	23(46)	0.34	46(33.1)	0.26
				0.54(0.31–0.94)		1.34(0.72–2.48)		0.77(0.50–1.20)
Recessive	TT + TC	228(96.2)	89(100)	Ref	50(100)	Ref	139(100)	Ref
	CC	9(3.8)	0	0.12	0	0.22	0	0.029
				0		0		0
Alleles (Dominant)	T	373(78.7)	155(87.08)	Ref	77(77)	Ref	232(83.45)	Ref
	C	101(21.3)	23(12.92)	0.01	23(23)	0.70	46(16.55)	0.11
				0.54(0.33–0.89)		1.10(0.65–1.84)		0.73(0.49–1.07)
Alleles	T	373(78.7)	155(87.08)	0.02				
				1.58(1.82–0.60)				
Recessive	C	101(21.3)	23(12.92)	Ref				

OR: odds ratio; CI: confidence interval; RA: rheumatoid arthritis; SLE: systemic lupus erythematosus

### The gene–gene interaction for both pre-miR499-rs374644 and pre-miR146- rs2910164 with susceptibility to RA and SLE

As shown in Table 4, in the analysis of gene–gene interaction, a significant reduction of RA risk was shown for the combination of TC and GG genotype in pre-miR499-rs374644 and pre-miR146-rs2910164 respectively, but not in SLE patients (TC+GG: *P*=0.01, OR=0.29, 95% CI=0.10-0.82).

**Table 4:** Association of different genotypic combinations with rheumatoid arthritis for miR499rs3746444 and miR146rs2910164

***Gene combination miR499- miR146***	***RA (89) n (%)***	***Control (237) n (%)***	***OR***	***(CI 95%)***	**P-*value***
TT-GG	27(30.34)	67(28.27)	-	-	Ref
TT-GC	34(38.20)	63(26.58)	1.33	(0.72–2.46)	0.34
TT-CC	5(5.62)	15(6.33)	0.82	(0.27–2.50)	0.74
TC-GG	5(5.62)	42(17.72)	0.29	(0.10–0.82)	0.01
TC- GC	17(19.10)	31(13.10)	1.36	(0.64–2.85)	0.41
TC- CC	1(1.12)	10(4.22)	0.24	(0.03–2.03)	0.28
CC-GG	0	4(1.68)	-	-	-
CC-GC	0	4(1.68)	-	-	-
CC-CC	0	1(0.42)	-	-	-

OR: odds ratio; CI: confidence interval; RA: rheumatoid arthritis

### Stratified analyses by sex with the pre-miR146-rs2910164 variant genotypes in arthritis patients and control participants

As shown in Table 5, the GC and GC+ CC genotypes compared with GG genotype were significantly associated with an increased risk of developing RA in females (*P*=0.007, OR=2.38, 95% CI=1.25–4.51; *P*=0.03, OR=1.95, 95% CI=1.05–3.60, respectively), but not with developing SLE.

**Table 5:** Stratified analyses by sex with miR146rs2910164 variant genotypes in arthritis patients and control participants

***Genotypes***	***Cases***	***Controls***	***OR (CI 95%)***	**P- *value***
GC/GG female	38/22	50/69	2.38(1.25–4.51)	0.007
GC/GG male	13/10	48/44	1.19(0.47–2.99)	0.70
GC+ CC/ GG female	43/22	69/69	1.95(1.05–3.60)	0.03
GC+ CC/ GG male	14/10	55/44	1.12(0.45–2.76)	0.80

OR: odds ratio; CI: confidence interval; RA: rheumatoid arthritis

## Discussion

The results of the present study showed that there is a risk of developing RA in carrying heterozygote GC versus GG genotype of pre-miR146-rs2910164. In contrast, analysis of pre-miR499-rs374644 showed a risk reduction for rheumatoid arthritis in TC + CC genotypes versus TT genotype and the C allele versus the T allele. Furthermore, in the analysis of gender, females carrying GC and GC + CC genotypes versus GG genotype were more likely to develop RA. Interestingly, individuals that carried both genotypes TC of miR499-rs374644 and GG genotype of pre-miRNA-146a rs2910164 were much less likely to be susceptible to RA.

No significant relationships were observed among polymorphisms of pre-miRNA-146a rs2910164 and pre-miRNA-499 rs3746444 with susceptibility to SLE.

Similar to the results of the present study, in the meta-analysis (10), no significant association was found between rs2910164 and SLE susceptibility in two Asian cohorts, one European cohort, and one Mexican cohort. In addition, studies were reported no significant association between rs2910164 and SLE susceptibility (11–14). In contrast, pre-miR146-rs2910164 G/C and pre-miR-499- rs3746444 A/G SNPs are related to SLE susceptibility but not to rheumatoid arthritis or Graves’ disease in the Mexican population (15).

Overall, so far, most of the studies conducted have not shown a potential impact for miR-146a and miR-499 in terms of SLE susceptibility. In the present study, the results of data analysis of RA are completely different with another study reported that the rs3746444 C allele of pre-miR-499 is linked to an increase in the risk of developing RA, but not rs2910164 of pre-miR-146a (9). The influence of ethnic differences (20) can be the cause of different outcomes of the two populations studied in Iran. But, in agreement with the results of the present study, two published meta-analyses have reported a significantly increased susceptibility to autoimmune disease in the carriers of the GC and GC+CC genotypes of pre-miR146 versus GG genotype and the carriers of GC genotype versus GG + CC genotype among the Caucasians and Asians, respectively (20,21). While the pre-pre-miR499-rs3746444 polymorphism is associated with RA risk in homozygote, recessive, and allele models, but not for pre-miR-146 in any genetic models (22). Highlighting the potential role of miRNAs as regulating the differentiation and function of immune cells indicates when phenotypical perturbations affect their expressive changes. Therefore, dysregulated microRNAs such as miR146a can contribute to the pathogenesis of autoimmune diseases, chronic inflammation, and malignancies (7,23–26). Pre-miR146a rs2710164 polymorphism modifies the expression level of miR146a (27), and thus has an aberrant regulatory effect on two key components (IL-1 receptor-associated kinase 1 (IRAK1) and (TRAF6) TNF receptor-associated factor 6) in the TLR4 signaling pathway (28). This evidence supports the pivotal role of rs2910164 as a functional SNP in susceptibility to RA. Specifically, the evaluation of *previous studies has shown that* the presence of the SNP rs2910164 located in miR-146a leads to the production of 2 isoforms miR-146a^*^G, and miR-146a^*^C. Therefore, 3 mature miRNAs are produced by GC heterozygotes including 1 from the leading strand and 2 from the passenger strand (miR-146a^*^G and miR-146a^*^C) and they differ from both GG and CC homozygotes (29–31).

In the present study, the increased risk of developing RA associated with GC genotype of miR-146 can be explained by the production of distinct miRNAs and the regulation of different target genes by heterozygotes compared to homozygotes. In other words, in GC heterozygote individuals, the production of three distinct miRNAs has inhibitory or aberrant effects that may be applicable to different target genes in RA patients.

The role of miRNA-499 in the pathogenesis of RA was associated with IL-17 as one of its targets up-regulated in synovium, synovial fluid and PBMC in RA patients. IL-17 is a pro-inflammatory cytokine that plays a crucial role in inducing the expression of TNF-a, IL-1b, IL-6, IL-23 and G-CSF (32–34). The relationship between the distributions of allele frequency with RA risk factors may explain the role of migraine pathogenesis in the disease. In this study, a significant difference in allele frequency of the T allele of rs3746444 of RA patients and the control group was observed, thus, suggesting a possible relationship between the activity of pre-miR499 with the T allele and the pathogenesis of RA. However, the C allele of rs3746444 had a protective role in RA patients in the present study. Interestingly, a two-fold increased risk of RA in the females in the present study points to a stronger influence of gender factors on autoimmune disease susceptibility. The high susceptibility of females can be attributed to the basic immune response that differs between men and women (4–6).

## Conclusion

Individuals with the least one C allele for rs3746444 of pre-miR-499 in interaction with the GG genotype rs29101164 of pre-miRNA-146a could have a protective effect on risk factor of RA.

In addition, women with GC+ CC genotype versus GG had a higher risk of developing RA than men.

## Ethical considerations

Ethical issues (Including plagiarism, informed consent, misconduct, data fabrication and/or falsification, double publication and/or submission, redundancy, etc.) have been completely observed by the authors.
